# Characteristic Cytokine Profiles of Aqueous Humor in Glaucoma Secondary to Sturge-Weber Syndrome

**DOI:** 10.3389/fimmu.2020.00004

**Published:** 2020-01-28

**Authors:** Cheng Peng, Yue Wu, Xuming Ding, Di Chen, Changjuan Zeng, Li Xu, Wenyi Guo

**Affiliations:** ^1^Department of Ophthalmology, Ninth People's Hospital, Shanghai Jiao Tong University School of Medicine, Shanghai, China; ^2^Shanghai Key Laboratory of Orbital Diseases and Ocular Oncology, Shanghai, China

**Keywords:** sturge-weber syndrome, secondary glaucoma, cytokines, intraocular pressure, aqueous humor

## Abstract

Patients with Sturge-Weber syndrome (SWS) are susceptible to ocular complications, and among them, glaucoma is one of the most frequent forms. In current study, we utilized multiplex human cytokine antibody array to simultaneously measure the concentration of 40 cytokines in aqueous humor (AH) of patients with SWS-induced glaucoma (SG), or from patients with senile cataract as controls. Compared with the control group, levels of interleukin (IL)-12p40, macrophage inflammatory protein (MIP)-1d, tumor necrosis factor-alpha (TNF-a), IL-5, IL-7, interleukin-6 receptor (IL-6R), and B lymphocyte chemoattractant (BLC) in AH were significantly higher in SG group. Samples from SG patients displayed significantly lower levels of MIP-1b, IL-6, MIP-1a, and monocyte chemoattractant protein (MCP)-1 than controls. Further analysis showed that IL-7, MIP-1a, TNF-a were positively correlated with intraocular pressure (IOP) in patients with early-onset SG. Moreover, IL-12p40 was negatively correlated with age in patients with SG. These cytokines may make contributions to the immunopathogenesis or progression of glaucoma in patients with SWS.

## Introduction

Sturge-Weber syndrome is a rare neurocutaneous disorder, which can be present sporadically and is characterized by vascular malformation usually composed of a unilateral facial port-wine stain (PWS) with ipsilateral deformed leptomeningeal vasculature as well as a choroidal hemangioma (1). Previous study showed that SWS is caused by the somatic mosaic mutation in GNAQ (2). Moreover, the severity of vascular malformation in SWS possibly depends on the timing of GNAQ mutation in the course of embryofetal development (3).

Inflammatory factors have been widely implicated in pathological process of many neurological disorders (4, 5). As for neurocutaneous syndrome, Neurofibromatosis-1(NF1), tuberous sclerosis complex (TSC) as well as SWS are the most common types, (6) and altered cytokine level has been previously reported in specimen of NF1 and TSC. Compared with healthy controls, the level of the inflammatory cytokines (IFN-γ, TNF-α, IL-6) in serum of NF1 patients are significantly higher, indicating the great importance of hyperinflammation in pathogenesis of NF1 (7). Similarly, significant proinflammatory cytokine expression in tuber of tuberous sclerosis complex (TSC) patients has been shown in either protein (8) or gene (9) level. Therefore, we assume that altered local cytokine profile can also be detected in SWS patients, which may contribute to pathogenesis and progression of the disease.

Secondary glaucoma is the major type of ocular complications in SWS, and its prevalence ranges from 30 to 70% in SWS patients (10). SG patients demonstrate a bimodal distribution. According to the onset age for glaucoma, 60% of them demonstrate an early onset in infancy and early childhood, whereas 40% of them demonstrate a late onset in late childhood and early adulthood (11). Pathogenesis of glaucoma in patients with SWS has been previously reported to be mainly associated with angle dysgenesis (10), elevation of episcleral venous pressure (EVP) (12), as well as an alteration in ophthalmic hemodynamics (13). Additionally, it is assumed that different pathogenesis may contribute to the bimodal-onset glaucoma. When it comes to early-onset glaucoma, angle dysgenesis plays a crucial role. On the other hand, in the late-onset form, elevated EVP that possibly results from dilated episcleral veins, is the main pathogenic factor (14). Nevertheless, the exact mechanism of elevated intraocular pressure (IOP) in SWS-induced glaucoma (SG) still remains unclear.

For glaucoma, morphological and functional changes in the trabecular meshwork (TM) are believed to be mainly responsible for increased resistance of AH outflow, and consequently lead to elevation of IOP (15, 16). Predominantly, these changes in the TM include the alteration in amount and the quality of the extracellular matrix (ECM) (17). Also, alteration in infiltrated immune cells, like neutrophils and macrophages, have been detected in the TM of various glaucomatous eyes (18, 19). In addition, it has been reported previously that endothelial cells of TM can secrete various factors and cytokines which play a pivotal role in regulation and modulation of ECM remodeling, and therefore affect the pathway of AH outflow (20, 21). Most recently, specific HSP-T cell response has been reported to play a pivotal role in IOP-independent glaucoma progression (22). Therefore, we hypothesize that immune factors may be the cause rather than the result of glaucoma in SWS patients.

To summarize, we speculated that changes in the cytokine-mediated signaling pathway can reflect the extent of glaucomatous development in SWS patients. Hence, detecting cytokine concentrations in the AH of SG patients may provide more insight into the local pathogenetic mechanism of this unique glaucoma subtype. In the present study, we utilized a Quantibody Human Inflammation Array 3 (Ray Biotech, Inc, Norcross, GA, USA) to investigate the cytokine profile in AH of SG patients.

## Materials and Methods

### Ethics Statement

This study was approved by the Institutional Review Board of Ninth People's Hospital, Shanghai Jiao Tong University School of Medicine, which followed the tenets of the Declaration of Helsinki. All methods were performed according to relevant guidelines and protocols, including any relevant details. Written informed consent was obtained for each participant. In case of underaged SG patients, written informed consent was obtained from parents or legal guardian.

### Subjects

In this cross-sectional study, diagnosis of SWS-induced glaucoma (SG) was based on general anesthesia examination for infant patients. IOP was not implemented as the decisive factor for final diagnosis, since the newborns presented lower level of IOP compared to adults. Close observation was given when an IOP above 21 mm Hg or apparent asymmetry between two eyes (above 6 mmHg) occurred. A cup-to-disc ratio (C/D) above 0.5, apparent asymmetry C/D between two eyes, corneal enlargement, and other corneal abnormalities, like Haab striae or corneal edema are strong diagnostic indicators of glaucoma (23). Patients undergoing routine surgery for uncomplicated, senile cataract were enrolled as controls.

For SWS patients, the clinical parameters (IOP, C/D, corneal diameters) were collected under general anesthesia before the glaucoma surgery. Agents used in the procedure of general anesthesia were stated below: sevoflurane or propofol for induction of anesthesia and either sevoflurane plus remifentanil or propofol plus remifentanil for maintenance of anesthesia. Measurement of IOP was carried out with an AccuPen Applanation Tonometer (Accutome, Inc, Malvern, Pennsylvania, USA). For all patients, a history of ocular or systemic inflammation stood for an exclusion criterion. For infant subjects, other systemic vascular malformations were ruled out except for PWS, and all babies followed the same routine vaccine scheme of China ensuring a similar vaccine-induced immunological background. Moreover, for enrolled individual patients, only one eye was included. The diagnosis of glaucoma, surgery and collection of AH sample and clinical data were all performed by the same doctor (W.Y. G.).

### Collection of Aqueous Humor

For both SG patients and controls, AH samples (50–100 μL) were collected at the start of surgery, by carrying out paracentesis with a 1.0 mL syringe and attached 30-gauge needle. After collection, all samples were instantly transferred and stored at −80°C for further cytokine analysis.

### Cytokine Antibody Array

The concentrations of 40 selected inflammatory cytokines was measured using a multiplex quantitative cytokines array (Ray Biotech, Inc, Norcross, GA, USA). The cytokines array was performed according to the manufacturer's guidelines. Samples were detected through use of a GenePix scanner (Axon Instruments, Inc, Foster City, CA, USA), and further analyzed with a GenePix Pro 6.0 software (Axon Instruments, Inc).

### Statistical Analysis

Principal component analysis (PCA) was carried out on cytokine concentrations after log transformation. Cluster analysis was carried out through use of 1-Pearson's correlation as well as Ward's hierarchical clustering. Pathway analysis was performed to investigate the significant pathways enriched by the differentially expressed cytokines according to Kyoto Encyclopedia of Genes and Genomes (KEGG) (http://www.genome.jp/kegg/). The PCA, cluster analysis and KEGG pathway analysis were performed through use of the R statistical package (http://www.r-project.org). Additional statistical analysis was performed using Prism for Windows software (Version 7.00, Graphpad Software, Inc.) and SPSS (Version 25.00, IBM SPSS Statistics, Inc.). The Shapiro-Wilk test was used to assess whether the data showed a normal distribution. Spearman's correlation analyses were used to evaluate the correlations between different cytokine levels, as well as correlations between concentrations and age, IOP, C/D and corneal diameter. Mann-Whitney U tests were used to evaluate the difference in cytokine levels between the groups (SG and control). Multiple linear regression was performed in subgroup analysis for adjustment of demographic differences.

Data were expressed as means and standard deviation (SD) or medians and interquartile range (IQR). A *P* < 0.05 was considered to be statistically significant, except for distinction of differentially expressed cytokine, for which a Bonferroni correction was applied. Because there were 40 different comparisons, a P-value less than 0.00125 (i.e., *P* = 0.05/40) was considered to be statistically significant with Bonferroni correction.

## Results

### Demographic Data

Overall, 22 SWS-induced glaucoma (SG) patients and 15 subjects with senile cataract were included in the present study. The demographic data including number of patients, age, sex, IOP, C/D, and corneal diameter, are summed up in Table 1. The median age of the SWS group and the control group were 2 years old and 77 years old, respectively (*P* < 0.001). No significant difference was observed in the sex distribution between the SG and the control groups (*P* = 0.7384).

**Table 1:** Demographic data.

	**SG**	**Control**	***P*-value**
Number of eyes	22	15	
Age [years; median (IQR)]	2 (0.52, 3.75)	77(71, 83.5)	<0.0001^*^
Sex (Female: Male)	11:11	9:6	0.7384^+^
IOP (mm Hg; mean ± SD)	20.41 ± 9.4	14.53 ± 3.38	***0.0119***^#^
C/D [median (IQR)]	0.725 (0.6125, 0.8)	n. a.	
Cornea diameter (mm; mean ± SD)	12.64 ± 0.92	n. a.	

Control, senile cataract eyes; SG, Sturge-weber syndrome induced glaucoma;

IOP, intraocular pressure; C/D, cup-to-disc ratio; IQR, interquartile range, shown as (25%, 75%);

n. a., not applicable;

* *Mann-Whitney U-test*,

# *Welch's t-test*,

+ *Fisher's Exact test*.

*P < 0.05 was considered significant (bold and italic)*.

### Cluster Analysis and Principal Component Analysis

**Figure 1:**
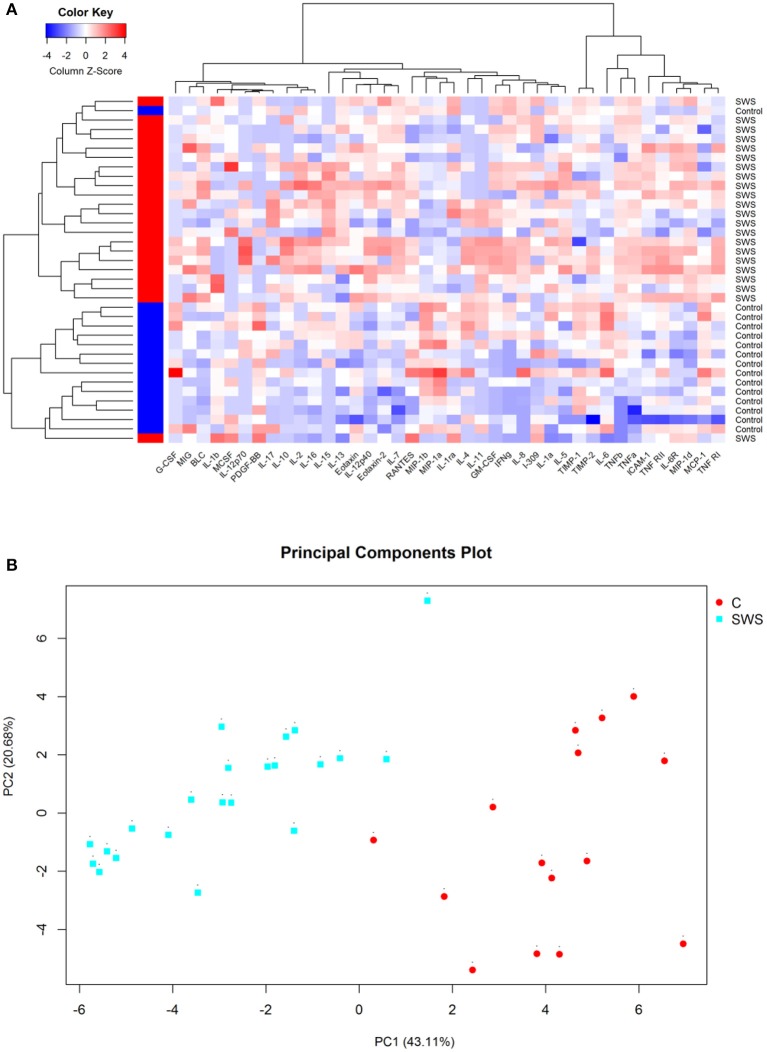
**(A)** Hierarchical clustering heat map of cytokines in aqueous humor. Cytokine concentrations are depicted as colors ranging from blue to white to red, presenting low, intermediate, and high concentration, respectively, according to the mean of that cytokine. The group of the samples is showed by the label on the right and by the colored bar on the left (red represents SWS and blue represents control). **(B)** Principal component analysis of aqueous humor samples, with the axes representing two principal components (PC1 and PC2). The fraction of variation that is displayed by each axis is noted in its label. The SWS samples are sharply discriminated from the control samples on the second principal component (PC2) that accounts for 20.68% of all variability.

Cluster analysis was utilized to obtain the overview of cytokine profile in the AH samples. The result showed that all AH samples can be separated into two major groups, and the two clusters isolated basically through the different expression of cytokines (Figure 1A). In addition, this analysis was confirmed by using principal component analysis (PCA). PCA data showed that the SWS group had a distinct component of different cytokines when compared to the control group (Figure 1B).

### Differences in the Cytokine Profile in the Aqueous Humor

**Figure 2:**
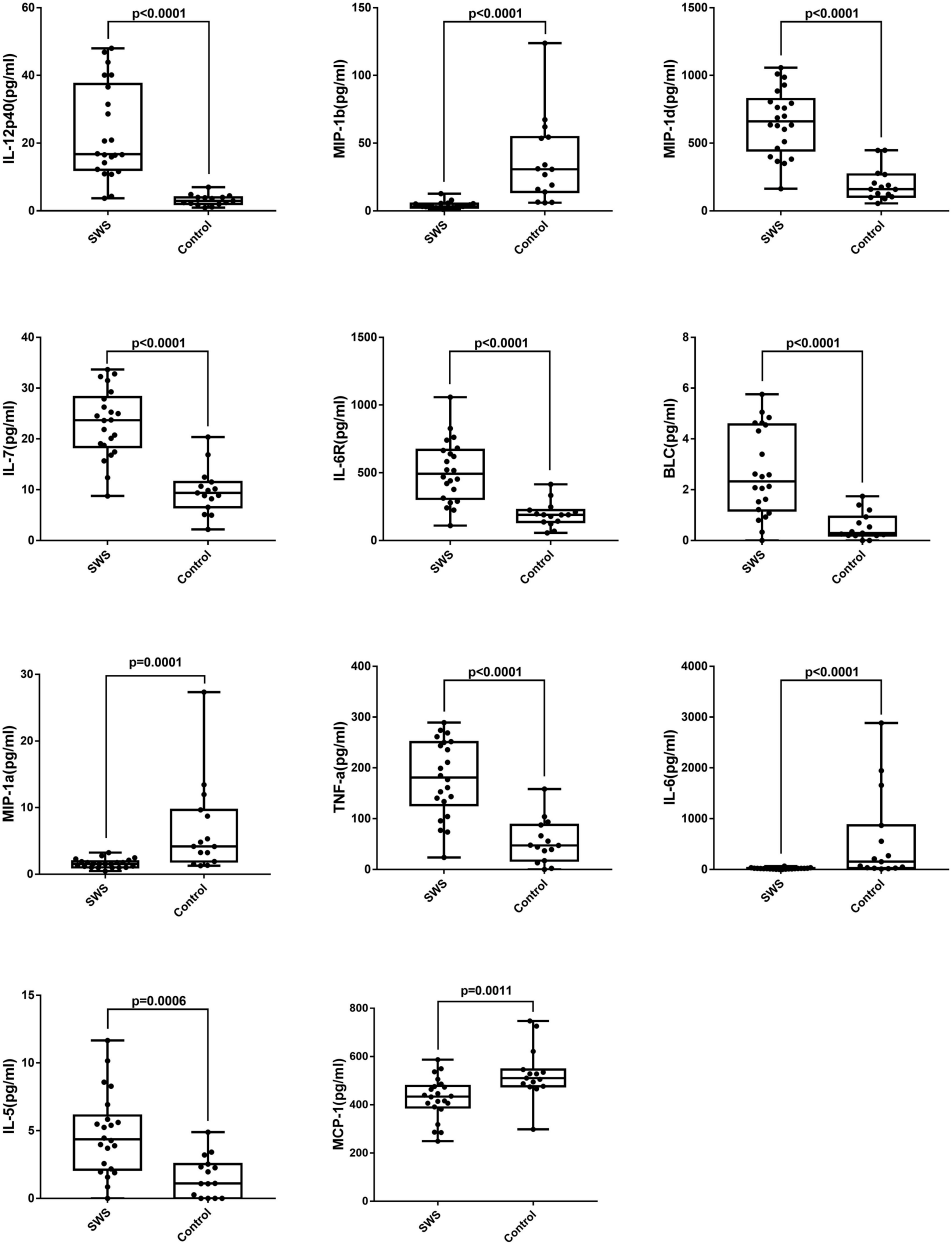
Box plots of IL-5, IL-6, IL-7, IL-12p40, IL-6R, MIP-1a, MIP-1b, MIP-1d, BLC, MCP-1, TNF-a in patients with SWS and senile cataract. Dots represent individual values (Mann-Whitney *U*-test with Bonferroni correction for multiple comparisons).

Table 2 shows the concentrations of 40 cytokines. Eleven cytokines were significantly expressed differently between two groups (Figure 2). Compared with controls, the SG group had a notably increased level of IL-12p40 (*P* < 0.0001), MIP-1d (*P* < 0.0001), IL-7 (*P* < 0.0001), IL-6R (*P* < 0.0001), BLC (*P* < 0.0001), IL-5 (*P* < 0.0001), and TNF-a (*P* < 0.0001). Moreover, the cytokine concentration of MIP-1b (*P* < 0.0001), IL-6 (*P* < 0.0001), MIP-1a (*P* = 0.0001), MCP-1 (*P* = 0.0011) were notably lower in the SG group.

**Table 2:** Concentrations of cytokines in SG and control groups.

	**SG**	**Control**	*P*-value^*^	**Min**	**Max**	**Detection rate**	
						**Control (%)**	**SG (%)**
**IL-12p40**	**23.07** **±** **13.92**	**3.18** **±** **1.58**	**<0.0001**	**0.4**	**20000**	**100.00**	**100.00**
**MIP-1b**	**4.01** **±** **2.52**	**36.77** **±** **31.6**	**<0.0001**	**1.4**	**2000**	**100.00**	**95.45**
**MIP-1d**	**659.92** **±** **240.51**	**195.19** **±** **119.51**	**<0.0001**	**1.3**	**20000**	**100.00**	**100.00**
**IL-7**	**23.06** **±** **6.69**	**9.74** **±** **4.56**	**<0.0001**	**13.8**	**8000**	**13.33**	**90.91**
**IL-6R**	**510.73** **±** **228.45**	**193.46** **±** **91.98**	**<0.0001**	**8.3**	**20000**	**100.00**	**100.00**
**BLC**	**2.67** **±** **1.71**	**0.55** **±** **0.54**	**<0.0001**	**0.2**	**4000**	**73.33**	**95.45**
**IL-6**	**19.58** **±** **14.32**	**584.71** **±** **883.53**	**<0.0001**	**19.5**	**4000**	**86.67**	**45.45**
**TNFa**	**179.67** **±** **75.3**	**54.23** **±** **42.72**	**<0.0001**	**86.2**	**4000**	**26.67**	**86.36**
**MIP-1a**	**1.61** **±** **0.66**	**6.81** **±** **6.88**	**0.0001**	**0.3**	**20000**	**100.00**	**100.00**
**IL-5**	**4.75** **±** **3.01**	**1.61** **±** **1.5**	**0.0006**	**7.6**	**8000**	**0.00**	**18.18**
**MCP-1**	**426.66** **±** **86.39**	**529.58** **±** **107.61**	**0.0011**	**8.8**	**4000**	**100.00**	**100.00**
ICAM-1	1953.87 ± 1490.18	693.09 ± 418.49	0.0033	1.3	200000	100.00	100.00
GM-CSF	12.11 ± 11.49	2.97 ± 4.3	0.0063	17.4	2000	0.00	22.73
IL-2	0.71 ± 0.54	0.27 ± 0.31	0.0076	1.8	4000	0.00	4.55
IFNg	12.84 ± 9.69	5.33 ± 7.58	0.0082	20	4000	6.67	22.73
TNFb	126.66 ± 82.28	54.73 ± 64.97	0.0091	210.9	40000	0.00	18.18
IL-8	7.27 ± 4.85	6.32 ± 15.47	0.0114	13.5	1000	6.67	13.64
IL-13	35.33 ± 29.28	14.68 ± 11.81	0.0116	46.5	2000	0.00	22.73
Eotaxin-2	21.7 ± 17.44	9.42 ± 5.65	0.0126	2.5	2000	86.67	95.45
IL-15	31.32 ± 43.98	3.21 ± 5.89	0.0172	43.3	8000	0.00	27.27
TIMP-1	5484.95 ± 486.46	5802.43 ± 524.14	0.0216	3	80000	100.00	100.00
MIG	2.36 ± 3.11	0.96 ± 1.93	0.0248	2.6	10000	6.67	22.73
TNF RI	686.03 ± 331.63	496.45 ± 225.33	0.1119	0.2	80000	100.00	100.00
I-309	5.13 ± 1.97	4.14 ± 2.14	0.1268	0.6	8000	100.00	100.00
IL-10	1.65 ± 1.97	0.48 ± 0.73	0.1369	6.4	8000	0.00	4.55
IL-16	0.98 ± 0.8	0.66 ± 0.4	0.2852	2.3	10000	0.00	13.64
Eotaxin	12.35 ± 8.73	8.8 ± 5.65	0.3342	1.2	8000	93.33	100.00
IL-1b	0.4 ± 0.52	0.3 ± 0.17	0.3929	0.2	2000	66.67	59.09
IL-11	15.85 ± 20.56	7.28 ± 9.82	0.4287	35.5	40000	0.00	18.18
IL-17	0.25 ± 0.26	0.16 ± 0.2	0.4351	2.4	8000	0.00	0.00
IL-4	5.32 ± 8.49	8.79 ± 13.41	0.4882	34.7	4000	6.67	0.00
TNF RII	266.79 ± 167.8	203.77 ± 101.05	0.4912	0.5	80000	100.00	100.00
IL-1ra	6.53 ± 8.11	5.41 ± 4.62	0.5107	0.8	4000	86.67	72.73
RANTES	17.63 ± 35.68	18.12 ± 36.8	0.5133	2	40000	73.33	77.27
G-CSF	0.84 ± 1.47	9.06 ± 28.03	0.5207	10.5	40000	6.67	0.00
MCSF	0.31 ± 0.52	0.27 ± 0.27	0.5211	0.3	8000	40.00	31.82
IL-12p70	0.09 ± 0.12	0.05 ± 0.06	0.5817	0.4	1000	0.00	0.00
PDGF-BB	0.05 ± 0.09	0.18 ± 0.09	0.6571	0.3	4000	6.67	4.55
IL-1a	2.91 ± 2.11	2.76 ± 1.89	0.7484	6.6	4000	0.00	9.09
TIMP-2	9073.98 ± 780.75	8942 ± 1036.59	0.9391	0.7	80000	100.00	100.00

Control, senile cataract eyes; SG, Sturge-weber syndrome induced glaucoma;

Mean ± SD; Cytokines: (pg/ml);

* Mann-Whitney U test;

*Values in boldface type indicate the 11 cytokines that were found to be significantly different between the two groups. Minimum and Maximum refer to the detection ranges for the 40 cytokines that were measured using the cytokine antibody arrays in accordance with the manufacturer's instructions. Detection rate shows the ratio of values within ranges to total*.

Table 3 presents the correlation among the eleven most differently expressed cytokines. Positive correlation was found between most of the eleven differently expressed cytokines, and there was no negative correlation between any of these cytokines.

**Table 3:** Correlation among 11 cytokines in SG patients.

**ρ/*P*-value**	**IL-12p40**	**MIP-1b**	**MIP-1d**	**IL-7**	**IL-6R**	**BLC**	**MIP-1a**	**IL-6**	**TNF-a**	**IL-5**	**MCP-1**
IL-12p40		0.2038	0.5325	0.5370	0.5743	0.8295	0.6951	0.5404	0.5449	0.3868	0.5460
MIP-1b	0.3629		0.1451	0.0762	0.3416	0.4602	0.6510	0.2016	0.0638	−0.1101	0.2242
MIP-1d	**0.0107**	0.5194		0.2072	0.6115	0.4647	0.3936	0.0073	0.4195	0.2208	0.1158
IL-7	**0.0100**	0.7360	0.3548		0.2513	0.3744	0.3665	0.5313	0.8069	0.6262	0.2772
IL-6R	**0.0052**	0.1197	**0.0025**	0.2593		0.7628	0.6849	0.1948	0.3732	0.1270	0.3642
BLC	**<0.0001**	**0.0312**	**0.0293**	0.0861	**<0.0001**		0.7775	0.3484	0.3416	0.2445	0.4082
MIP-1a	**0.0003**	**0.0010**	0.0700	0.0935	**0.0004**	**<0.0001**		0.3190	0.3348	0.0604	0.5426
IL-6	**0.0094**	0.3683	0.9741	0.0109	0.3850	0.1121	0.1479		0.5731	0.4816	0.6217
TNF-a	**0.0087**	0.7779	0.0519	**<0.0001**	0.0871	0.1197	0.1277	**0.0053**		0.5776	0.3010
IL-5	0.0754	0.6257	0.3235	**0.0018**	0.5732	0.2728	0.7894	**0.0232**	**0.0049**		−0.0536
MCP-1	**0.0086**	0.3159	0.6080	0.2116	0.0956	0.0593	**0.0091**	**0.0020**	0.1735	0.8126	

*Correlation coefficient (ρ) and P-values for each pair of cytokines are calculated by Spearman's correlation test*.

*P < 0.05 was considered significant (bold)*.

Table S2 demonstrates the result of subgroup analysis. In order to adjust the cytokine level with age, we used multiple linear regression in both SG group(infants) and cataract group(elders) separately. The data demonstrated that age did not influence the cytokine level except for IL12-p40 and IL-6R in the SG group, and the demographic data did not affect any cytokine level in the cataract group. Therefore, the result suggested that the difference in the cytokine expression is mainly due to the disease itself instead of age-caused bias.

### Correlation of Cytokine Levels With Clinical Parameters

In order to explore the direct association between cytokine levels and clinical parameters, we performed spearman correlation test in the SG group. Except for the negative correlation between concentration of IL-12p40 and age of patients, there is no statistically significant correlation between cytokine level and clinical parameters, such as age, IOP, C/D and corneal diameter (*P* = 0.0046; Table S1).

**Figure 3:**
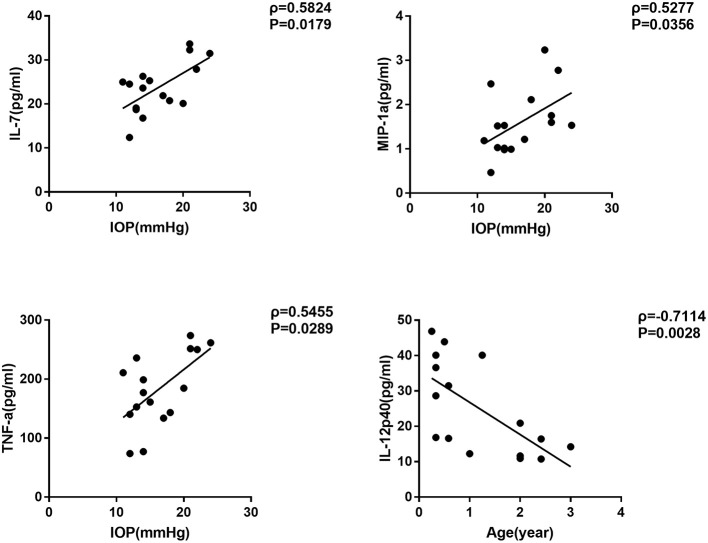
Scatterplots showing the correlation of four selected cytokines with IOP and age in early-onset SG subgroup. Correlations between IOP and, respectively: IL-7 (ρ = 0.5824, *P* = 0.0179), MIP-1a (ρ = 0.5277, *P* = 0.0356), TNF-a (ρ = 0.5455, *P* = 0.0289). Additionally, Correlations between age and IL-12p40 (ρ = −0.7114, *P* = 0.0028) (Spearman's correlation test). The solid line represents the linear regression line.

We further selected the patients who had surgery before the age of four as the candidates of early-onset SG group, and carried out the same correlation analysis (Table 4). Interestingly, we noticed that the concentration of three cytokines were remarkably correlated with IOP in the early-onset SG patients (IL-7, MIP-1a and TNF-a; ρ = 0.5824, 0.5277, 0.5455; *P* = 0.0179, 0.0356, 0.0289, respectively; Figure 3). Additionally, concentration of IL-12p40 was negatively correlated with age in early-onset SG patients (ρ = −0.7114; *P* = 0.0028; Figure 3).

**Table 4:** Correlation of cytokines with age, IOP, C/D, or corneal diameter in early-onset SG group.

	**Age**		**IOP**		**C/D**		**Corneal diameter**	
	**Correlation coefficient**	***P*-value**	**Correlation coefficient**	***P*-value**	**Correlation coefficient**	***P*-value**	**Correlation coefficient**	***P*-value**
IL-12p40	−0.7114	***0.0028***	0.3548	0.1776	0.1509	0.5770	0.2023	0.4492
MIP-1b	−0.1682	0.5336	0.3636	0.1662	−0.1154	0.6704	−0.4167	0.1090
MIP-1d	−0.3572	0.1744	0.4095	0.1153	−0.2130	0.4283	−0.0604	0.8240
IL-7	−0.2828	0.2886	0.5824	***0.0179***	0.2219	0.4088	0.2038	0.4458
IL-6R	−0.2024	0.4522	0.4671	0.0681	0.0133	0.9610	0.0876	0.7459
BLC	−0.4554	0.0763	0.4080	0.1167	0.1021	0.7068	0.2461	0.3552
MIP-1a	−0.4866	0.0559	0.5277	***0.0356***	0.1923	0.4755	−0.0619	0.8196
IL-6	−0.2962	0.2654	0.3577	0.1737	−0.0118	0.9653	0.3564	0.1748
TNF-a	−0.2247	0.4027	0.5455	***0.0289***	0.2101	0.4349	0.0151	0.9573
IL-5	−0.1369	0.6131	0.3237	0.2213	0.0399	0.8832	0.1842	0.4915
MCP-1	−0.2456	0.3593	0.3030	0.2539	0.1109	0.6825	0.1389	0.6054

IOP, intraocular pressure; C/D, cup-to-disc ratio;

*The correlation coefficient and P-values for cytokines and age, IOP, C/D, or corneal diameter were calculated by Spearman's correlation test for early-onset SG eyes. The tests demonstrate correlation between cytokine levels (IL-12p40, IL-7, MIP-1a and TNF-a) and clinical parameters (age and IOP)*.

*P < 0.05 was considered significant (bold and italic)*.

### KEGG Pathway Analysis

**Figure 4:**
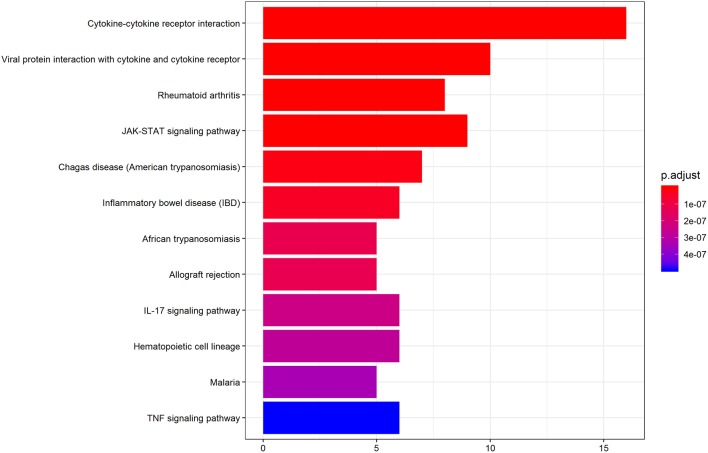
KEGG pathway analysis indicates the significant pathways enriched by differentially expressed cytokines in AH of patients. Top 12 pathways for the differentially expressed cytokines are displayed. The x-axis shows the amounts of cytokines associated with KEGG pathway. The color ranging from blue to red presents different level of adjusted *p*-value.

We further performed KEGG pathway analysis to investigate the significant pathways enriched by the differentially expressed cytokines. The pathways enriched by the differential cytokines in aqueous humor samples are shown in Figure 4. The results demonstrated that the differential cytokines were significantly enriched in mainly four pathways including cytokine-cytokine receptor interaction (*P* = 1.33E-20), viral protein interaction with cytokine and cytokine receptor (*P* = 5.23E-14), JAK-STAT signaling pathway (*P* = 2.34E-10) and rheumatoid arthritis(RA) (*P* = 1.45E-10).

## Discussion

In this study, we utilized the cytokine antibody array method to find out the cytokine profile of the AH in SWS-induced glaucoma (SG) and age-related cataract patients. We found that the cytokine profiles were significantly different between the two groups. The level of IL-12p40, MIP-1d, IL-7, IL-6R, BLC, TNF-a, IL-5 were elevated in the SG samples compared to the samples of the cataract patients. However, the concentration of MCP-1, IL-6, MIP-1b, MIP-1a were lower in the SG group. Furthermore, we showed the cytokine level of four cytokines (IL-12p40, IL-7, MIP-1a, and TNF-a) were correlated with clinical parameters (age and IOP) in the early-onset SG patients.

Previous studies revealed that significantly different aqueous humor inflammatory cytokine expression can appear in different types of glaucomatous eyes. In primary open angle glaucoma (POAG) eyes, the level of IL-12, IL-6, TNF-α, IL-8, IFN-γ were noted to be remarkably raised (24–26). In pseudoexfoliation glaucoma (PEG) eyes, an increase of CCL13, CCL15, CCL22, CCL24, CXCL13, CXCL16, IL-4, IL-6, IL-8, and IL-16 were observed (27, 28). For uveitic glaucomatous eyes, IL-6, IL-8, VEGF, CCL2, and TNF-α were detected to be higher compared to the controls (29). Also, in the neovascular glaucoma (NVG) patients, increased level of IL-6, IL-8, PDGF, CCL2, and TNF-α in AH were previously reported (30). In our study, there was a significant difference in the inflammation-related cytokine expression between the SG and cataract patients, presenting a specific cytokine profile in the aqueous of SWS patients, indicating that cytokine-mediated inflammation was involved in the development of SG.

IL-12 is a proinflammatory cytokine which promote Th1 cell differentiation and have a pivotal role in T cell-mediated response. It is composed of two subunits (IL12-p40 and IL-12p35), and is mainly secreted from activated antigen-presenting cells (APCs), for example, dendritic cells (DCs) (31). The level of IL-12p40 has been reported to be increased in many conditions of autoimmune disorders, such as psoriasis, rheumatoid arthritis (RA), Crohn's disease (32) and uveitis (33). In addition, inflammatory and angiogenic processes are recognized as the crucial factors to the development of diabetic retinopathy (DR) and diabetic macular edema (DME). Moreover, patients with DR or DME have a higher concentration of IL-12 in the AH when compared with controls (34). Consistently, it has been previously shown that DME patients have lower levels of IL-12 after treatment (35). In the current study, the level of IL-12p40 in the AH of SG patients were notably higher compared with controls, indicating that the inflammatory and angiogenic function of IL-12 may contribute to pathogenesis of SG.

MIP-1d(CCL15) is secreted by various cells (36) in normal condition and is elevated in autoimmune disorders such as RA (37) and asthma (38). MIP-1d can stimulate the migration and differentiation of the chemotactic endothelial cell, and it has a pivotal role in angiogenesis (39). Moreover, MIP-1d was reported to be important in tumor progression by recruiting suppressive monocytes to facilitate immune escape and angiogenesis (40). Our results showed that MIP-1d level was significantly higher in SWS group. Considering the important role of vascular malformation in the facial, leptomeningeal, choroidal lesions of SWS, we hypothesize that ocular (scleral and/or choroidal) angiogenesis may contribute to the pathogenesis in ocular hypertension and RGC degeneration of SG. If proved in future animal experiments or clinical trials, MIP-1d could be of use to the treatment of SWS-induce choroidal hemangioma and glaucoma.

MCP-1(CCL2), MIP-1α (CCL3), and MIP-1β (CCL4) act as typical chemoattractant that is involved in the inflammatory reaction by recruiting different immune cells to the injured area (41, 42). Additionally, they were previously reported to be increased in many pathological conditions of ocular inflammation, including glaucoma (43) and polypoidal choroidal vasculopathy (PCV) (44). Interestingly, in the present study, we found decreased MCP-1, MIP-1α, and MIP-1β concentrations in the eyes of SG patients when compared with the cataract eyes, indicating a conceivable ‘atypical inflammation’ in the aqueous humor during the disease progression. The explanation for the discrepancy may be as follows: on the one hand, previous studies showed MCP-1 was notably elevated in AH in the contralateral eye following the first-eye cataract surgery (45). In our study, among the 15 cataract patients, 9 patients had already had phacoemulsification for the contralateral eyes before the aqueous samples were taken, which may induce the relatively higher concentration of chemokines detected in the cataract group. On the other hand, the normal trabecular meshwork endothelial cells constitutively secrete chemokines, which maintain homeostasis of the outflow of the aqueous humor (46). Hence, the constitutive secretion of these chemokines may be affected by the damaged trabecular meshwork endothelial cells in the SWS-induced glaucomatous progression.

Tumor necrosis factor-alpha (TNF-a) is a proinflammatory cytokine with pleiotropic effects on various cell types. Enhanced TNF-a expression have been previously reported in AH, trabecular meshwork, retina and optic nerve of clinical glaucomatous specimen, which is consistent with our present finding (19, 25, 47–49). IL-6 is also a proinflammatory cytokine that promotes inflammatory responses and is involved in wound-healing and leucocyte recruitment (50, 51). Many studies of different types glaucoma have shown an increased level of IL-6 in aqueous humor (24, 29, 30). However, in the current study, our result showed a decreased level of IL-6 in SWS patients. An explanation of the inconsistency might be that cytokines are expressed in a complicated regulatory pattern, which can be affected by a variety of microenvironmental stimuli, and might therefore differ quite considerably in another disease subtype or in another course of disease. It is noteworthy that, in our study, we observed significantly higher levels of IL-6R in the AH of SG patients. It has been shown that soluble IL-6ra (sIL-6Ra) can interact with IL-6, further constituting the IL-6/sIL-6Ra complex, which can bind to glycoprotein-130 on cells and then induce IL-6 trans-signaling (52). Additionally, IL-6 trans-signaling can increase IL-6 activity under inflammatory environment and to further prevent the apoptosis of T cells, which probably play a crucial role in the exacerbation and the prolongation of autoimmune disease processes (53–55). Currently, biologic agents of anti-TNF-α and anti-IL-6 receptor antibodies are widely used for uveitis treatment with satisfying clinical outcomes (56–58). Therefore, there is a possibly important role for these cytokine-related inflammations to play in the pathogenesis of SG, which could provide the therapeutically potential target for SG in the near future.

Previous studies reported significant correlations between cytokines and clinical parameters of glaucoma. In POAG and PEG patients, TGF-β, IL-8, and SAA were positively correlated with pre-operative IOP, and IL-6 was positively correlated with age (59) There was also a report that found IL-8, IP-10, CCL2, and CCL4 were correlated with IOP in POAG and NVG eyes (43). Moreover, the positive correlation of G-CSF level between IOP has been reported in AH of acute primary angle-closure (APAC) patients (60). In the current study, initially, there were no cytokines correlated with IOP in SG patients. Considering that bimodal-onset glaucoma may be involved in different pathogenesis, we further classified the SWS group into the early-onset (age of surgery <4 years) and late-onset (age of surgery > 4 years) groups. Surprisingly, we noted that the level of IL-7, MIP-1a, and TNF-a were positively correlated with the preoperative IOP in eyes of early-onset SG. Coincidently, a recent study of rheumatoid arthritis (RA) showed that the high responsiveness to IL-7 stimulation together with the synergy of IL-7 and M1-promoting factors (e.g., MIP-1b, TNF-a) can enhance the expression of joint myeloid IL-7R and further exacerbate arthritis (61). Consistently, our KEGG analysis also suggested that RA and SG may have the same cytokine microenvironment in common (Figure 4). Additionally, we found IL12-p40 was significantly negatively correlated with age in SWS group, which implicated that the IL-12 mediated-inflammation may be associated with the development of early-onset SG. Taken together, the result indicated that the intricate cytokine networks may play a pivotal role in pathogenesis and progression of early-onset SG, although the specific mechanism of interactions among these cytokines needed to be further validated.

The current research did have several limiting factors. First, a relatively small sample size may lead to statistical bias. However, the small sample size may result from the strict selection criteria ensuring the same immunological background of our subjects, as well as the rarity of disease. Second, the age difference between two groups was large, which was partially responsible for the differences of cytokine profile. Even though we were fully aware that age-matched non-glaucoma infant with SWS may be the ideal choice for control group, they were not available in the current study for the ethical issues. Nevertheless, after statistical adjustment, we could at least draw a tentative conclusion that altered cytokine levels is mainly caused by the pathology of disease rather than the age-associated bias. Third, no functional analysis conducted to elucidate the exact mechanism between these altered cytokines and IOP elevation. Since the very small volume of samples and unidentified cell type or animal model in SG pathogenesis, we can hardly further investigate the immunopathogenesis of altered cytokine profile in SG. Further studies utilizing intraocular tissue specimen of SG patients or developed animal models may be conducive to exploring the potential roles of cytokine-related inflammation and angiogenesis in the pathological process of SG.

In conclusion, the present study firstly showed the characteristic cytokine profiles of aqueous humor in patients with SG. Additionally, the intraocular levels of a wide variety of cytokines were related to clinical parameters, such as age and IOP. Further investigations including animal experiments and clinical trials are necessary to explore the relative importance and mutual mechanism of these cytokines.

## Data Availability Statement

The datasets generated for this study are available on request to the corresponding author.

## Ethics Statement

The studies involving human participants were reviewed and approved by Institutional Review Board of Ninth People's Hospital, Shanghai Jiao Tong University School of Medicine. Written informed consent to participate in this study was provided by the participants' legal guardian/next of kin.

## Author Contributions

CP and WG designed the study. WG, CP, LX, XD, and CZ collected aqueous humor samples. CP, YW, and DC performed the measurements and data analysis. CP, YW, and WG wrote the manuscript. All authors have read and critically revised the manuscript.

### Conflict of Interest

The authors declare that the research was conducted in the absence of any commercial or financial relationships that could be construed as a potential conflict of interest.

## Supplementary Material

The Supplementary Material for this article can be found online at: https://www.frontiersin.org/articles/10.3389/fimmu.2020.00004/full#supplementary-material
