# Supplementary material for: Characteristic Cytokine Profiles of Aqueous Humor in Glaucoma Secondary to Sturge-Weber Syndrome
Source: Front Immunol. 2020 Jan 28;11:4. doi: 10.3389/fimmu.2020.00004 (PMC7008723; doi:10.3389/fimmu.2020.00004)
Supplement: Supplementary file 1 [file Data_Sheet_1.docx]

|  | Age | | IOP | | C/D | | Corneal Diameter | |
| --- | --- | --- | --- | --- | --- | --- | --- | --- |
|  | Correlation coefficient | P-value | Correlation coefficient | P-value | Correlation coefficient | P-value | Correlation coefficient | P-value |
| IL-12p40 | -0.5810 | ***0.0046*** | -0.0362 | 0.8728 | -0.0216 | 0.9240 | 0.1677 | 0.4558 |
| MIP-1b | 0.0363 | 0.8725 | 0.3164 | 0.1513 | -0.0392 | 0.8625 | -0.1566 | 0.4864 |
| MIP-1d | -0.1487 | 0.5091 | 0.1500 | 0.5052 | -0.1273 | 0.5725 | 0.1828 | 0.4155 |
| IL-7 | -0.3302 | 0.1334 | 0.1393 | 0.5365 | -0.0278 | 0.9021 | 0.1298 | 0.5647 |
| IL-6R | 0.0045 | 0.9840 | 0.3866 | 0.0755 | 0.0699 | 0.7573 | 0.3545 | 0.1055 |
| BLC | -0.4023 | 0.0635 | 0.0787 | 0.7278 | -0.0551 | 0.8075 | 0.2474 | 0.2669 |
| MIP-1a | -0.1702 | 0.4489 | 0.3068 | 0.1649 | 0.1733 | 0.4406 | 0.1106 | 0.6241 |
| IL-6 | -0.2417 | 0.2785 | 0.1466 | 0.5150 | -0.0955 | 0.6726 | 0.1112 | 0.6223 |
| TNFa | -0.2060 | 0.3578 | 0.1834 | 0.4139 | -0.0068 | 0.9760 | 0.1356 | 0.5472 |
| IL-5 | -0.3308 | 0.1327 | -0.0266 | 0.9064 | -0.0875 | 0.6986 | 0.0215 | 0.9242 |
| MCP-1 | -0.2820 | 0.2036 | 0.0469 | 0.8355 | -0.0335 | 0.8823 | 0.0291 | 0.8977 |

**Table S1. Correlation of cytokines with age, IOP, C/D, or corneal diameter in SG patients.**

IOP, intraocular pressure; C/D, cup-to-disc ratio;

The correlation coefficient and P-values for cytokines and age, IOP, C/D, or corneal diameter were calculated by Spearman’s correlation test for SG eyes. The tests demonstrate the between cytokine level of IL12-p40 and age.

P < 0.05 was considered significant (bold and italic).

|  | Age-SG | | Age-Control | |
| --- | --- | --- | --- | --- |
|  | Correlation coefficient | P-value | Correlation coefficient | P-value |
| IL-12p40 | -0.634 | ***0.014*** | 0.044 | 0.899 |
| MIP-1b | -0.257 | 0.440 | 0.015 | 0.968 |
| MIP-1d | -0.614 | 0.054 | -0.116 | 0.751 |
| IL-7 | -0.392 | 0.233 | -0.399 | 0.237 |
| IL-6R | -0.762 | ***0.012*** | -0.101 | 0.778 |
| BLC | -0.620 | 0.053 | -0.360 | 0.232 |
| MIP-1a | -0.315 | 0.346 | 0.277 | 0.450 |
| IL-6 | -0.276 | 0.403 | -0.064 | 0.859 |
| TNFa | -0.399 | 0.217 | -0.520 | 0.132 |
| IL-5 | -0.207 | 0.535 | -0.515 | 0.127 |
| MCP-1 | -0.288 | 0.394 | -0.241 | 0.506 |

**Table S2. Subgroup analysis by using multiple linear regression for age adjustment.**

Control, senile cataract eyes; SG, Sturge-weber syndrome induced glaucoma;

The correlation coefficient and P-values for cytokine levels and demographic data (age, sex and IOP) were assessed by multiple linear regression analysis for two subgroups (SG and cataract). The data shows except for cytokine level of IL-12p40 and IL-6R, cytokine levels are not influenced by age.

P < 0.05 was considered significant (bold and italic).
